# Glycemic Control with Thiazolidinedione Is Associated with Fracture of T2DM Patients

**DOI:** 10.1371/journal.pone.0135530

**Published:** 2015-08-28

**Authors:** Hsin-Hung Chen, Ming-Hwarng Horng, Su-Yin Yeh, I-Ching Lin, Chih-Jung Yeh, Chih-Hsin Muo, Fung-Chang Sung, Chia-Hung Kao

**Affiliations:** 1 School of Public Health, Chung Shan Medical University, Taichung, Taiwan; 2 School of Medicine, Chung Shan Medical University, Taichung, Taiwan; 3 Division of Metabolism & Endocrinology, Changhua Christian Hospital, Changhua, Taiwan; 4 Division of Metabolism & Endocrinology, Nantou Christian Hospital, Nantou, Taiwan; 5 Division of Critical Care Medicine, Department of Internal Medicine, Changhua Christian Hospital, Changhua, Taiwan; 6 Changhua Christian medical foundation, Yuanlin Christian Hospital, Changhua, Taiwan; 7 Department of Healthcare Administration, Asia University, Taichung, Taiwan; 8 Department of Family Medicine, Changhua Christian Hospital, Changhua, Taiwan; 9 Education and Research on Geriatrics and Gerontology, Chung Shan Medical University, Taichung, Taiwan; 10 Management Office for Health Data, China Medical University Hospital, Taichung, Taiwan; 11 College of Medicine, China Medical University, Taichung, Taiwan; 12 Graduate Institute of Clinical Medical Science and School of Medicine, College of Medicine, China Medical University, Taichung, Taiwan; 13 Department of Nuclear Medicine and PET Center, China Medical University Hospital, Taichung, Taiwan; Rensselaer Polytechnic Institute, UNITED STATES

## Abstract

**Objective:**

Diabetes is a common diseases and a major problem worldwide. Diabetic osteopathy might be elevated in diabetic patients and is usually caused by bone fracture. Several diabetes medications, such as thiazolidinediones (TZDs), could lead to increased risks of fracture.

**Methods:**

We used the nationwide database to identified 32466 patients who had developed type 2 diabetes from 2000 to 2010 as the diabetic cohort and, from that group, we selected 3427 diabetic patients who had developed bone fracture to survey the possible risk factors, includng commonly used diabetes medication.

**Results:**

We found that TZDs might present increased risks for fracture in patients who used it for an extended period (7 to 730 days before the index date), especially in female patients younger than 64 years old, for whom the risk was elevated from a 1.74- to a 2.58-fold odds ratio.

**Conclusions:**

We recommend that clinics follow up with non-osteoporotic female patients younger than 64 years old who are using TZDs, to avoid the associated risks of fracture.

## Introduction

Diabetes is a worldwide public health crisis and in 2011 the WHO mentioned diabetes as one of the 4 critical non-communicable diseases in the world. Diabetes affects the skeletal system in a condition called diabetic osteopathy [1], which can increase the risk of bone fracture in diabetic patients, and may reduce bone healing after fracture [2,3]. Thiazolidinediones (TZDs) are one of the medications prescribed for glycemic control. The PROactive [4] and ADOPT [5] studies indicated that TZDs can improve end-organ sensitivity to the effects of insulin, reduce triglycerides, and increase high-density lipoprotein levels. The net effect of TZDs is that they can lower an excess risk of macro-vascular or micro-vascular disease in diabetic patients. In general clinical practice, TZDs has been challenged due to some side effects such as bone fractures, congestive heart failure, weight gain, and cancer. However, many recent publications have suggested that TZDs can increase the risk of fracture, through various mechanisms, in diabetic patients. Furthermore, there were many studies to evaluate the associations between TZDs and fractures. Because of mixed results from these previous studies, current guidelines in the world could not offer use to rules for limiting TZDs use in order to avoid fracture risk or prevent bone fracture in high risk group. We assert that TZDs are valuable medications in the treatment of insulin-resistant patients or diabetic patients with chronic kidney disease (CKD), and therefore our goal is to find the diabetic group for whom the prescription of TZDs is free from risk of fracture.

## Materials and Methods

### Data source

We used the National Health Insurance Database (NHIRD), which was obtained from the National Health Program, administered by the Taiwanese Bureau of National Health Insurance and maintained by the Taiwanese National Health Research Institute. This program covered over 99% of the Taiwanese population in 2010 and was established on March 1, 1995. The NHIRD includes the medical claims and information of each beneficiary from 1996 to 2010. We used the Longitudinal Health Insurance Database 2000 (LHID2000) and the catastrophic illness patient registry (CIPR) in this nested case-control study. The LHID2000 contains 1 million beneficiaries, randomly selected from the original beneficiaries in the 2000 registry. The details of the LHID2000 have been used in prior papers [6].

### Study Subjects

From the claims data for patients from 2000 to 2010, we identified 32466 patients with newly diagnosed type 2 diabetes (T2DM, ICD-9-CM code 250.00, 250.02) and excluded those younger than 20 years of age (n = 308) or those who had a fracture history before the date of diagnosis with type 2 diabetes (n = 5559). Patients with newly diagnosed bone fractures (ICD-9-CM code 800–829) were included as the DM cohort. The study group consisted of 3427 T2DM patients with fractures from the DM cohort and the date of diagnosis was defined as the index date. The comparison group consisted of patients randomly selected from the remaining T2DM patients without a bone fracture history. The index date in the comparison group was randomly assigned after the T2DM date. For each bone fracture case, we randomly selected 1 comparison person, frequency matching for sex, age (every 5-year span), gender, DM-year, and index-year.

### Included variables

The baseline comorbidity history for each subject included end-stage renal disease (ESRD, ICD-9-CM codes585 from CIPR), stroke (ICD-9-CM codes, 434.91 from inpatient claims), ischemic heart disease (IHD, ICD-9-CM codes, 410–414), hypertension (ICD-9-CM codes, 401–405), and osteoporosis (ICD-9-CM codes, 733.0).

### T2DM medication

Both in-patient and out-patient use of T2DM medications are reimbursed by the NHI, which allows for the validation of T2DM in the population. The T2DM medications in this study included thiazolidinediones (TZD), glyburide, glimepiride, gliclazide, chlorproapmide, metformin, acarbose, nateglinide, repaglinide, and insulin, which were prescribed before the index date.

### Data Availability Statement

All data and related metadata were deposited in an appropriate public repository. The data on the study population that were obtained from the NHIRD (http://w3.nhri.org.tw/nhird//date_01.html) are maintained in the NHIRD (http://nhird.nhri.org.tw/). The NHRI is a nonprofit foundation established by the government. These data were released by the NHIRD for research uses. Every interested researcher is able to obtain the data in the same way that we did.

### Ethics Statement

The NHIRD encrypts patient personal information to protect privacy and provides researchers with anonymous identification numbers associated with relevant claims information, including sex, date of birth, medical services received, and prescriptions. We excluded all individually identifying or patient demographic information. Therefore, the patient consent is not required to access the NHIRD. This study was approved by the Institutional Review Board (IRB) of China Medical University (CMU-REC-101-012). The IRB specifically waived the consent requirement.

### Statistical Analysis

The demographic characteristics and prevalence of comorbidities were compared between the study and comparison group. The differences were examined using the *X* ^2^ test for categorical variables, and the *t*-test for continuous variables. The odds ratio and 95% confidence interval (CI) for bone fractures were used in the logistic regression. The multivariate model was used as a control for age, gender, and comorbidity, which are shown to be significantly different between the 2 groups, in Table 1. Because there were different in the distribution of TZD and repaglinide used between patients with and without fracture, we estimated the joint effect for fracture between TZD and repaglinide. We also assessed the association between the bone fracture and the periods of T2DM medication use before the index date. All analyses were performed using the SAS statistical package (version 9.2; SAS Institute Inc., Cary, NC, USA). A 2-tailed *P* value <.05 was considered statistically significant.

**Table 1:** Demographics between patients with and without fracture.

	Fracture				
	No (N = 3427)		Yes (N = 3427)		
	n	%	n	%	p-value
**Sex**					0.99
Female	1811	52.8	1811	52.8	
Male	1616	47.2	1616	47.2	
**Age, year**					0.99
20–54	1037	30.3	1037	30.3	
55–64	878	25.6	878	25.6	
65–74	827	24.1	827	24.1	
≥ 75	685	20.0	685	20.0	
Mean (SD)	62.3	(13.1)	62.6	(13.3)	0.30^†^
**Medical history**					
ESRD	12	0.35	31	0.90	0.004
Stroke	302	8.81	360	10.5	0.02
IHD	995	29.0	1060	30.9	0.09
Hypertension	2319	67.7	2331	68.0	0.76
Osteoporosis	323	9.43	468	13.7	<0.0001
**DM medication**					
TZD	415	12.1	494	14.4	0.005
Glyburide	1141	33.3	1183	34.5	0.29
Glimepiride	1020	29.8	1045	30.5	0.51
Gliclazide	1293	37.7	1316	38.4	0.57
Glipizide	636	18.6	677	19.8	0.21
Chlorpropamide	7	0.20	9	0.26	0.62
Metformin	1925	56.2	1997	58.3	0.08
Acarbose	422	12.3	457	13.3	0.21
Nateglinide	92	2.68	99	2.89	0.61
Repaglinide	290	8.46	365	10.7	0.002
Insulin	3083	90.0	3079	89.9	0.87

Chi-square test,

^†^ t-test

## Results

We identified 32466 patients who had developed T2DM from 2000 to 2010 as the DM cohort and, from that group, we selected 3427 T2DM patients who had developed bone fracture, and excluded the patients who had a fracture history. When we compared patients with and without fracture, the mean ages of 62.6 (SD = 13.3) and 62.3 (SD = 13.1) years showed no significant difference (t test, *P* = .30), as shown in Table 1. Additionally, the rates of females with and without fractures were equal (52.8%). The comorbidities involved in ESRD were: stroke, ischemia heart disease, hypertension, and osteoporosis. However, ESRD (*P* = .004), stroke (*P* = .02), and osteoporosis (*P*< .0001) showed significant effects on the presence of fractures, as listed in Table 1. The TZDs (*P* = .005) and repaglinide (*P* = .002) showed a significant association between patients with and without fracture (Table 1).

The joint effect analysis for fracture between TZDs and repaglinide was shown in Table 2. Among all the patients, those who were presented with the combined treatment of TZDs and repaglinide showed an elevated risk for fracture of 1.51 (95% CI, 1.13–2.01) in all T2DM patients, which was adjusted for age, gender, ESRD, stroke, and osteoporosis. Furthermore, the female T2DM patients showed similar results, in which the risk of developing fracture was less when only using TZDs as 1.29 (95% CI, 1.04–1.59) or more when using TZDs and repaglinide as 1.68 (95% CI, 1.14–2.48), as shown in Table 2.

**Table 2:** Joint effect between TZD and Repaglinide by gender.

DM medication	All	Men	Women
**All**			
Neither	1.00 (reference)	1.00 (reference)	1.00 (reference)
TZD	1.16 (0.99–1.36)	1.05 (0.83–1.33)	1.29 (1.04–1.59)*
Repaglinide	1.16 (0.96–1.41)	1.13 (0.85–1.49)	1.19 (0.91–1.56)
Both	1.51 (1.13–2.01)**	1.35 (0.88–2.06)	1.68 (1.14–2.48)*
**Non- Osteoporosis**			
Neither	1.00 (reference)	1.00 (reference)	1.00 (reference)
TZD	1.18 (1.00–1.40)	1.03 (0.81–1.30)	1.36 (1.08–1.73)*
Repaglinide	1.18 (0.95–1.46)	1.12 (0.84–1.50)	1.24 (0.91–1.69)
Both	1.44 (1.06–1.94)*	1.29 (0.84–1.98)	1.61 (1.05–2.46)*
**Osteoporosis**			
Neither	1.00 (reference)	1.00 (reference)	1.00 (reference)
TZD	1.04 (0.64–1.70)	3.78 (0.41–34.6)	0.97 (0.58–1.61)
Repaglinide	1.10 (0.66–1.82)	1.18 (0.36–3.88)	1.07 (0.61–1.87)
Both	2.40 (0.86–6.64)	--	2.15 (0.76–6.07)

Adjusted for age, gender, ESRD, stroke and osteoporosis

The odds ratio of overall and female T2DM patients for developing fracture during various periods indicated that, if using TZDs from 7 days to more than 1 year, the odds ratio decreased from 1.36 to 1.26 in all patients, and from 1.79 to 1.41 in women (Table 3), compared to subjects without TZD treatment in the same period. Using repaglinide in all patients was shown to increase the odds ratio of fracture among various periods, as shown in Table 3.

**Table 3:** Odds ratio for fracture among different period of drug used by gender.

	All	Men	Women
**Overall**			
TZD	1.19 (1.03–1.37)*	1.08 (0.87–1.33)	1.31 (1.08–1.59)**
Repaglinide	1.20 (1.01–1.41)*	1.16 (0.91–1.48)	1.22 (0.97–1.54)
**Within 7 days before index date**			
TZD	1.36 (1.10–1.69)**	1.01 (0.73–1.38)	1.79 (1.32–2.41)***
Repaglinide	1.02 (0.75–1.39)	1.07 (0.68–1.67)	0.95 (0.62–1.47)
**Within 7–30 days before index date**			
TZD	1.32 (1.07–1.63)**	1.02 (0.75–1.39)	1.66 (1.24–2.22)***
Repaglinide	1.04 (0.77–1.40)	1.02 (0.66–1.57)	1.04 (0.69–1.57)
**Within 30–90 days before index date**			
TZD	1.27 (1.03–1.55)*	1.03 (0.76–1.39)	1.53 (1.15–2.03)**
Repaglinide	1.09 (0.82–1.44)	1.10 (0.73–1.65)	1.06 (0.72–1.56)
**Within 90–365 days before index date**			
TZD	1.23 (1.02–1.48)*	1.02 (0.78–1.34)	1.45 (1.13–1.86)**
Repaglinide	1.17 (0.91–1.50)	1.18 (0.82–1.69)	1.15 (0.82–1.63)
**Within 365–730 days before index date**			
TZD	1.26 (1.04–1.53)*	1.11 (0.84–1.48)	1.41 (1.08–1.84)*
Repaglinide	0.98 (0.76–1.27)	0.91 (0.63–1.32)	1.05 (0.73–1.51)

Adjusted for age, gender, ESRD, stroke and osteoporosis

The odds ratio for fracture varied for female patients, according to various periods of drug use by various age groups. The 20- to 54-year age group showed decreased risks of fracture during the period of TZD treatment, which was below the 90 days observed for females without TZD treatment during the same time period. The age group of 55 to 64 years showed that the highest risk of fracture is among those using TZD within 30 days of the index date (1.74 to 1.77; Table 4). Using repaglinide might significantly elevate the risks for fracture from 2.17 to 2.61 for those between 65 to 74 years of age in time periods within 30 to 365 days before the index date (Table 4).

**Table 4:** Odds ratio for fracture among different period of drug used in women.

	Age group, year			
	20–54	55–64	65–74	≥ 75
**Overall**				
TZD	1.74 (1.17–2.58)**	1.17 (0.82–1.66)	1.18 (0.81–1.73)	1.09 (0.68–1.75)
Repaglinide	1.18 (0.68–2.06)	1.04 (0.66–1.64)	1.83 (1.19–2.79)**	0.97 (0.62–1.52)
**Within 7 days before index date**				
TZD	2.58 (1.38–4.84)**	1.77 (1.05–2.99)*	1.31 (0.77–2.22)	1.35 (0.50–3.68)
Repaglinide	0.83 (0.28–2.42)	0.57 (0.22–1.46)	1.72 (0.82–3.62)	0.82 (0.33–2.07)
**Within 7–30 days before index date**				
TZD	2.10 (1.16–3.80)*	1.74 (1.06–2.85)*	1.25 (0.74–2.11)	1.34 (0.51–3.52)
Repaglinide	1.01 (0.39–2.83)	0.73 (0.30–1.76)	1.83 (0.91–3.70)	0.75 (0.33–1.74)
**Within 30–90 days before index date**				
TZD	1.87 (1.06–3.30)*	1.35 (0.86–2.14)	1.37 (0.80–2.34)	1.21 (0.43–3.45)
Repaglinide	0.84 (0.33–2.18)	0.74 (0.32–1.72)	2.17 (1.06–4.41)*	0.73 (0.33–1.62)
**Within 90–365 days before index date**				
TZD	1.63 (0.97–2.73)	1.37 (0.89–2.10)	1.24 (0.78–1.99)	1.53 (0.73–3.24)
Repaglinide	1.20 (0.53–2.71)	0.75 (0.38–1.48)	2.61 (1.30–5.21)**	0.77 (0.37–1.58)
**Within 365–730 days before index date**				
TZD	1.40 (0.82–2.40)	1.42 (0.89–2.25)	1.24 (0.76–2.03)	1.65 (0.81–3.39)
Repaglinide	0.82 (0.36–1.87)	0.84 (0.41–1.71)	2.01 (0.96–4.20)	0.87 (0.43–1.76)

Adjusted for ESRD, stroke and osteoporosis

## Discussion

After fracture, bone healing usually follows 3 stages, namely, the inflammatory stage, reparative stage, and remodeling stage [7]. Fracture callus formation may be slow, and fracture healing times may be prolonged by up to 87% in diabetic patients [8]. The TZDs work through stimulation of the PPAR-γ receptor which can decrease the peripheral insulin resistance and hepatic glucose output of diabetic patients [9]. However, through the PPAR-γ receptor, TZDs can affect the bone marrow pluripotent mesenchymal stem cells, which can differentiate into adipocytes or osteoblasts, but the stimulation may cause preferential differentiation into adipocytes over osteoblasts [10]. In 2 randomized, controlled trials in humans receiving rosiglitazone, levels of bone formation markers such as osteocalcin and procollagen type I N-terminal propeptide [11, 12] declined significantly. Numerous recent studies have suggested that TZDs use the PPAR-γ receptor to suppress the bone anabolic signaling pathway, which can decrease the activity of Wnt [13]. This process results in decreased osteoblast numbers, and decreased formation of new vessels [14]. Another mechanism whereby TZDs affect fracture healing time is through their inhibitory effects on estrogen and androgen biosynthesis [13].

A recent study from ACCORD BONE [15] suggested that intensive glycemic control is not associated with fractures or falls. The intensive glycemic treatment group had greater exposure to all anti-diabetic drugs than the standard group. In the intensive glycemic treatment group, 92% of participants were prescribed TZDs compared with 58% in the standard group, and daily doses of TZDs were higher in the intensive than the standard glycemic treatment group [16]. Previous clinical trials indicate that TZD use doubles the risk of fracture in women, but has no effect in men [17]. The comparison of the glycemic groups in the ACCORD BONE study [15] suggests that, because of a lack of adequate equipment to detect a 20% increase in the relative rate of fracture, TZD use (or even higher TZD exposure in the standard group), makes it is difficult to draw conclusions about associations between TZD use and fracture risk.

In our analysis, we found the effect of TZDs to be similar to those found by other studies. When we adjusted the factors (Table 2), we found that TZDs still have effects in non-osteoporotic women, but have no effect in osteoporotic women. Our study showed that TZDs could increase the risk of fracture within 7 days in women, and the effects remained for 64 year-old women. Compare to recent researches, one study mentioned that fracture risk was independent of age and there was no clear association with duration of TZDs exposure [18]. But in the other study, prolonged users of TZDs could increase the risk of fracture [19]. Because adequate evidence to discuss the results in our study is lacking, we list some possible explanations. First, TZDs do not have a higher effect on diabetic patients with osteoporosis in Asia than elsewhere. Second, TZDs do not have more influence on women older than 64 years because of the sex hormone factors such as estrogen and androgen [13]. Third, fracture is more frequently affected by other diabetic complications such as hypoglycemia with non-sulfonylureas (Tables 1 and 4), diabetic neuropathies, impaired vision by diabetic retinopathy, and lower limb amputations, such as diabetic foot [20,21], rather than TZD use.

In our study, We also found that repaglinide could play a role for fracture for older diabetic patients. However, One study for Repaglinide/troglitazone combination therapy did not mentioned the risk of fracture [22]. Another study for combination treatment with non-sulfonylureas and TZDs also showed no side effects with the risk of fracture [23]. Repaglinide was a kind of non-sulfonylureas which was prescribed for flexible prandial glucose regulation in order to avoid hypoglycemia [24]. Hypoglycemia could be a role of falling and it could cause fracture. Possible explanations for our finding were mentioned below. First, repaglinide was only lesser hypoglycemia than common sulfonylureas [25] and in order to avoid hypoglycemia in older patients, repaglinide was prescribed for these older group. Second, repaglinide could be allowed for diabetic patients with chronic kidney diseases instead of other sulfonylureas.

## Conclusions

Previous studies have demonstrated the risk between TZDs and fracture in diabetic groups, but we propose that the risk is relative for diabetic patients in Asia. The relatively safe group for diabetic patients to use TZDs in our study is osteoporotic female patients, older than 64 years in Asia.

### Limitations

First, we could not collect the complete data which could be the risks of osteoporosis or fracture such as calcium supplement, sun exposure and other nutrition status. Secondly, image data such as X-ray finding or bone marrow density also could not be collected. Third, TZDs could cause all site of fracture, we could not list the detail sites of fracture.
